# Severe Hypercalcemia and Acute Kidney Injury as Initial Manifestations of Multisystem Sarcoidosis in an Adolescent With Type 1 Diabetes: A Case Report

**DOI:** 10.7759/cureus.105121

**Published:** 2026-03-12

**Authors:** Yuxin Wang, Meredith Ott, Rachel Ekdahl, Amanda Costa

**Affiliations:** 1 Department of Family and Preventive Medicine, University of Arkansas for Medical Sciences, Little Rock, USA; 2 Department of Pediatrics, University of Arkansas for Medical Sciences, Little Rock, USA; 3 Department of Pediatrics, Division of Pediatric Hospital Medicine, Arkansas Children's Hospital, University of Arkansas for Medical Sciences, Little Rock, USA

**Keywords:** acute kidney injury, corticosteroid, multidisciplinary evaluation, multiorgan involvement, pediatric sarcoidosis, severe hypercalcemia, type 1 diabetes

## Abstract

Pediatric sarcoidosis is a rare noninfectious granulomatous disease that can affect any organ. Diagnosis is challenging due to a lack of specific tests and highly variable symptoms that can resemble infectious processes or malignancy.

We report a 16-year-old Caucasian girl with well-controlled type 1 diabetes mellitus (T1DM) who presented with 10 days of nausea, vomiting, and generalized abdominal pain and was incidentally found to have severe hypercalcemia and acute kidney injury (AKI). Further testing revealed high 1,25-dihydroxyvitamin D (calcitriol) and angiotensin-converting enzyme (ACE) levels, with suppressed parathyroid hormone (PTH) and normal PTH-related peptide (PTHrP). An extensive infectious workup was negative. Imaging demonstrated generalized lymphadenopathy, raising concern for lymphoma or granulomatous disease. Lymph node biopsy showed non-caseating granulomas consistent with sarcoidosis. She received corticosteroids and steroid-sparing agents, resulting in normalization of calcium and symptom resolution.

This case illustrates a rare, high-risk pediatric sarcoidosis with multiorgan involvement and severe hypercalcemia, emphasizing the need for biopsy confirmation and multidisciplinary evaluation in distinguishing it from infection or malignancy.

## Introduction

Sarcoidosis, also known as Besnier-Boeck-Schaumann disease, is a systemic granulomatous disorder that primarily affects the lungs and lymphatic system. The cause remains unclear, but the current hypothesis is that a combination of genetic susceptibility and environmental exposure activates CD4+ T cells and macrophages, leading to granuloma formation [1]. Although sarcoidosis is well described in adults, it is rare in children, and the literature on pediatric sarcoidosis is limited. Two distinct forms of childhood sarcoidosis have been described. There is an early-onset disease in children younger than five years, characterized by a triad of rash, arthritis, and uveitis. The second is a later-onset form in adolescents that tends to be adult-like pulmonary disease [2]. Because of its diverse presentation, lack of specific tests, and overlap with infection or malignancy [3], pediatric sarcoidosis remains a diagnosis of exclusion, requiring histologic confirmation after other causes are ruled out [4].

Certain forms of sarcoidosis are considered high-risk, including treatment-resistant pulmonary disease, cardiac sarcoidosis, neurosarcoidosis, and multiorgan involvement [5,6]. These phenotypes are associated with greater morbidity and mortality and are often underrecognized, especially in pediatric patients [5,7]. Early identification is critical to prevent complications and guide appropriate therapy.

Sarcoidosis may also mimic or coexist with malignancy, a phenomenon known as sarcoidosis-lymphoma syndrome. This association may occur when sarcoidosis precedes, coincides with, or develops after lymphoma [8]. The relationship is most often seen in adults and typically involves Hodgkin’s lymphoma [8]. Both can present with non-caseating granulomas and lymphadenopathy, making the distinction difficult on imaging and histology. The proposed link may involve shared immune dysregulation and chronic antigenic stimulation, which predispose to lymphoid proliferation.

Besides its association with lymphoma, sarcoidosis may also coexist with autoimmune diseases such as type 1 diabetes mellitus (T1DM). Both conditions are immune-mediated and share Th1/Th17 pathway dysregulation with increased pro-inflammatory cytokines, including TNF-α, IL-1, and IL-6 [9], suggesting a common immunologic background rather than a causal link. While sarcoidosis has been associated with other autoimmune endocrinopathies, concurrence with T1DM is exceptional.

## Case presentation

A 16-year-old Caucasian girl with well-controlled T1DM presented with 10 days of nausea, vomiting, and intermittent generalized abdominal pain. She also reported unintentional weight loss of 10 kg over six months and a mild headache for a month. She denied diarrhea, fever, rash, joint pain, or night sweats. There was no recent travel, tuberculosis exposure, or use of over-the-counter medications, including calcium supplements. Initial laboratory investigations showed elevated creatinine (1.7 mg/dL) and severe hypercalcemia (14.2 mg/dL), with other labs unremarkable. Physical examination was significant for diffuse abdominal tenderness and bilateral cervical adenopathy without overlying skin changes. Hypercalcemia workup showed suppressed parathyroid hormone (PTH), normal PTH-related peptide (PTHrP), elevated calcitriol, and elevated angiotensin-converting enzyme (ACE) levels (Tables 1-2).

**Table 1:** Key Basic Laboratory Findings at Presentation The laboratory results demonstrated severe hypercalcemia (calcium 14.2 mg/dL) with elevated 1,25-dihydroxyvitamin D (105 pg/mL) and a suppressed PTH level. Normocytic anemia was noted, a feature frequently observed in chronic inflammatory conditions like sarcoidosis. PTH: parathyroid hormone; PTHrP: parathyroid hormone-related peptide; CRP: C-reactive protein; WBC: white blood cell; RBC: red blood cell; ESR: erythrocyte sedimentation rate; LDH: lactate dehydrogenase

Category	Laboratory Test	Result	Reference Range
Basic workup	Calcium (total)	14.2 mg/dL	8.5-10.7 mg/dL
	Phosphorus	2.3 mg/dL	2.7-4.7 mg/dL
	Creatinine	1.7 mg/dL	0.6-1.2 mg/dL
	Calcium, ionized	1.73 mmol/L	1.1-1.3 mmol/L
	25-hydroxyvitamin D	30.5 ng/mL	20-65 ng/mL
	1,25-dihydroxyvitamin D	105 pg/mL	19.9-79.3 pg/mL
	PTH	<6.3 pg/mL	12.0-65.0 pg/mL
	PTHrP	0.8 pmol/L	<4.2 pmol/L
	Uric acid	7.0 mg/dL	3.5-5.9 mg/dL
	CRP	<5 mg/L	0-9.9 mg/L
	WBC	3.6 x 10^3^/µL	4.5-13.0 x 10^3^/µL
	RBC	3.12 x 10^6^/µL	4.1-5.1 x 10^6^/µL
	Hemoglobin	8.5 g/dL	12.0-16.0 g/dL
	ESR	65 mm/hr	0-20 mm/hr
	LDH	112 U/L	100-190 U/L
	Haptoglobin	195 mg/dL	44-215 mg/dL

**Table 2:** Key Immunology Workup Findings During Hospitalization Immunology workup demonstrated elevated ACE at 165 U/L (reference: 18-101 U/L), increased lysozyme at 6.91 µg/mL (reference: <4.5 µg/mL), and mildly elevated sIL-2RA at 2517 pg/mL (reference: 214-1910 pg/mL). ANA testing showed a homogeneous pattern with a titer of 1:320. ACE: angiotensin-converting enzyme; ANA: antinuclear antibody; Anti-dsDNA: anti-double-stranded DNA antibody; ANCA: anti-neutrophil cytoplasmic antibody; β2-glycoprotein IgG/IgM: beta-2 glycoprotein immunoglobulin G; sIL-2RA: soluble interleukin-2 receptor alpha; IgG: immunoglobulin G; IgM: immunoglobulin M; C3: complement component 3; C4: complement component 4; Anti-Smith: anti-Smith antibody; Anti-RNP: anti-ribonucleoprotein antibody; Anti-SSA: anti-Sjögren’s-syndrome-related antigen A; Anti-SSB: anti-Sjögren’s-syndrome-related antigen B; TTG-IgA: tissue transglutaminase immunoglobulin A

Category	Laboratory Test	Result	Reference Range
Immunology workup	ACE	165 U/L	18-101 U/L
	ANA pattern	Homogenous	-
	Anti-dsDNA	12 IU	<24 IU
	Myeloperoxidase antibody	0	0-19 AU/mL
	ANCA titer	<1:20	<1:20
	β2-glycoprotein IgG/IgM	Negative	-
	Cardiolipin IgG/IgM	Negative	-
	Lysozyme	6.91 µg/mL	<4.5 µg/mL
	sIL-2RA	2517 pg/mL	214-1910 pg/mL
	IgG	2093 mg/dL	528-2190 mg/dL
	IgG 4	77 mg/dL	2-170 mg/dL
	C3	142 mg/dL	70-206 mg/dL
	C4	23.1 mg/dL	11-61 mg/dL
	Anti-Smith	5	0-40 AU/mL
	Anti-RNP	5	0-19 AU/mL
	Anti-SSA	1	0-40 AU/mL
	Anti-SSB	1	0-40 AU/mL
	TTG-IgA	0.3	0.0-6.9 U/mL
	IgM	166 mg/dL	40-230 mg/dL

**Figure 1:**
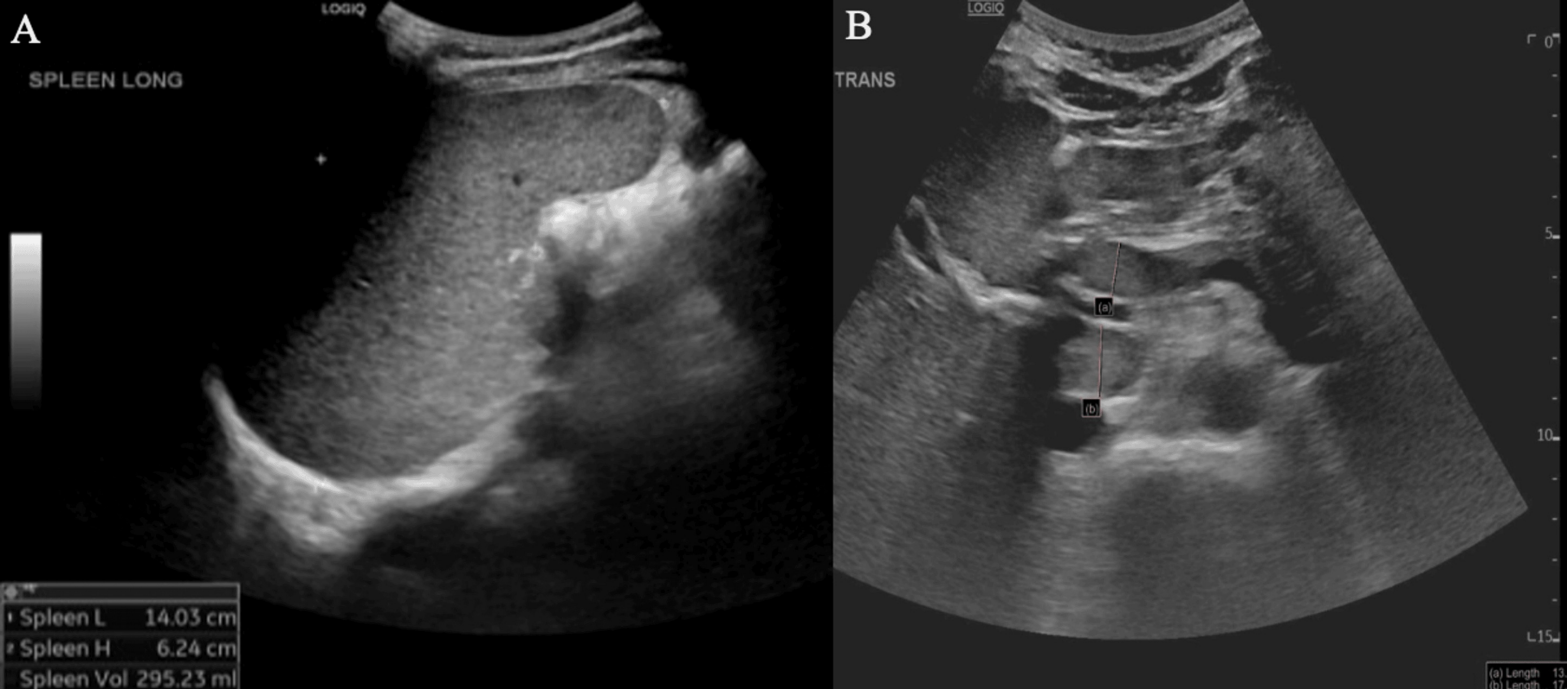
Abdominal Ultrasound (A) Abdominal ultrasound was remarkable for splenomegaly and periportal adenopathy. (B) The spleen measures 14.03 cm in length, with a volume of 295.23 mL. Periportal lymph nodes are seen, measuring up to 13 mm (node a) and 17 mm (node b) in the short axis.

Given the severity of hypercalcemia with associated acute kidney injury (AKI), nephrology and endocrinology were consulted, and they agreed with aggressive IV hydration (at 1.5× maintenance rate). Calcium levels decreased as oral hydration improved, and creatinine normalized with IV hydration. However, headaches and nausea persisted throughout admission, and calcium levels rebounded as fluids were decreased to a maintenance rate. Hypercalcemia workup had excluded primary hyperparathyroidism, and a decision was made to pursue further imaging to assess for secondary causes of hyperparathyroidism, such as granulomatous disease versus malignancy. Abdominal ultrasound demonstrated periportal adenopathy and splenomegaly (Figure 1).

**Figure 2:**
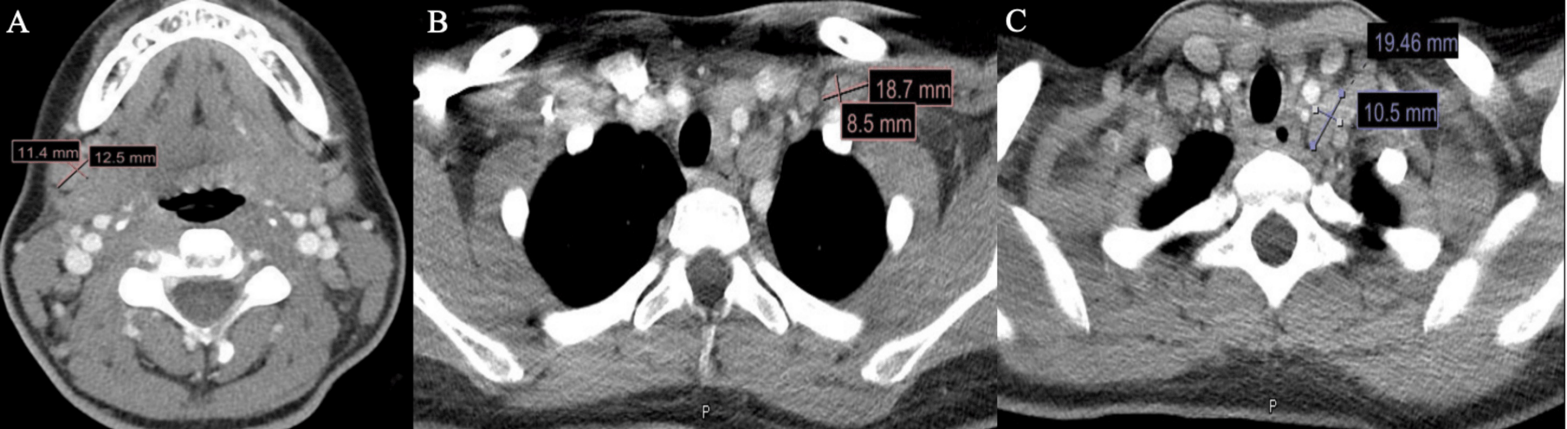
Computed Tomography (CT) of the Neck CT of the neck demonstrating cervical lymphadenopathy. A few prominent bilateral cervical and submandibular lymph nodes are visible, with the largest measuring 12.5 × 11.4 mm in the right submandibular level (labeled in A), 18.7 × 8.5 mm in the left supraclavicular level (labeled in B), and 19.46 × 10.5 mm in the left lower cervical level (labeled in C).

**Figure 3:**
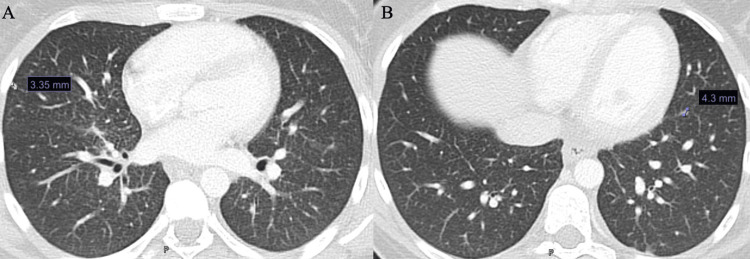
Computed Tomography (CT) of the Chest CT of the chest showing small pulmonary nodules. One 3.35 mm nodule is present in the right middle lobe, and one 4.3 mm nodule is seen along the left lung fissure (labeled in A and B).

**Figure 4:**
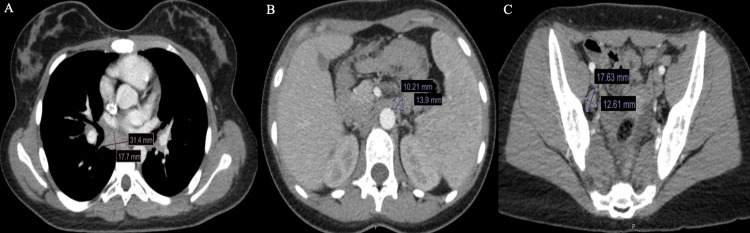
Computed Tomography (CT) of the Abdomen and Pelvis Multiple large mediastinal lymph nodes are seen. The largest lymph node measures 31.4 × 17.7 mm at the subcarinal level (A). Another lymph node measures 13.9 × 10.21 mm at the preaortic level (B), and a third measures 17.63 × 12.61 mm at the right external iliac level (C).

Subsequent computed tomography (CT) imaging of the neck, chest, abdomen, and pelvis revealed diffuse cervical, mediastinal, hilar, retroperitoneal, and iliac lymphadenopathy, along with parotid gland enlargement with intraparotid lymph nodes, multiple small pulmonary and splenic nodules, and a small lytic lesion of the sternal manubrium (Figures 2-4).

After the results of imaging, hematology, oncology, and rheumatology were consulted. Extensive autoimmune evaluations were negative, except for elevated lysozyme and soluble IL-2 receptor alpha (Table 2). Due to her exposure to cats, dogs, and chickens, infectious disease was consulted, and tests for *Bartonella*, *Histoplasma*, *Blastomyces*, *Toxoplasma*, and *Mycobacterium tuberculosis* were performed - all of which were negative (Table 3).

**Table 3:** Key Infectious Workup Findings During Hospitalization TB: tuberculosis; HIV: human immunodeficiency virus; EBV: Epstein-Barr virus; CMV: cytomegalovirus; PCR, polymerase chain reaction; IgG: immunoglobulin G; IgM: immunoglobulin M All infectious evaluations, including bacterial, viral, fungal, and parasitic testing, were negative.

Category	Laboratory Test	Result	Reference Range
Infectious workup	TB Quantiferon	Negative	Negative
	HIV	Negative	Negative
	EBV PCR	Not detected	-
	CMV PCR	Not detected	-
	Adenovirus PCR	Not detected	-
	*Bartonella* IgG	<1:64	<1:64
	*Bartonella* IgM	<1:16	<1:16
	*Histoplasma* antigen	Not detected	Not detected
	*Histoplasma* antibody	<1:8	<1:8
	*Blastomyces* antigen/antibody	Not detected	Not detected
	*Toxoplasma* IgG/IgM	Negative	-

**Figure 5:**
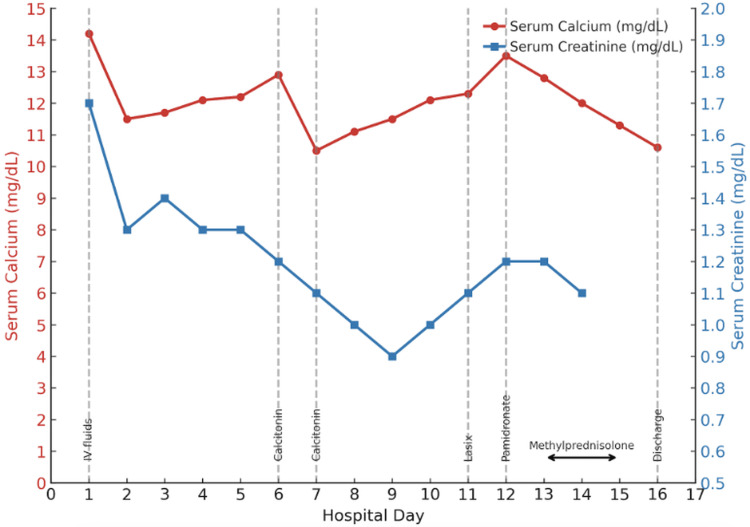
Serum Calcium and Creatinine Trends With Treatment The patient initially presented with severe hypercalcemia and AKI. Intravenous hydration led to partial improvement, while subsequent administration of calcitonin provided only transient calcium reduction. A single dose of pamidronate on day 12 resulted in sustained normalization of calcium levels. Methylprednisolone initiated on day 13 contributed to the stabilization of both calcium and renal function. AKI: acute kidney injury

Rheumatology suggested a formal ophthalmologic examination due to concern for sarcoidosis, which was normal, without evidence of uveitis. The patient received a course of calcitonin, which improved calcium but worsened her nausea and was discontinued. The furosemide trial had a minimal effect on calcium levels. A single dose of pamidronate was given, which normalized calcium and relieved symptoms (Figure 5). Zoledronic acid was avoided due to AKI.

**Figure 6:**
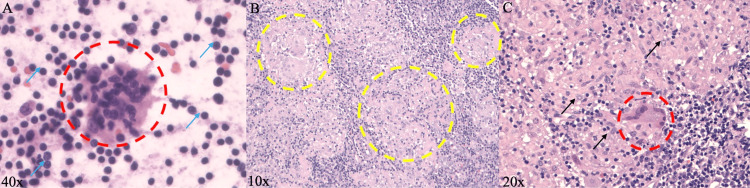
Pathology Result of Lymph Node Biopsy (A) Touch preparation of a lymph node at 40× magnification demonstrating multinucleated giant cells (red circle) within a background of normal lymphocytes (blue arrows). (B) H&E-stained section of a lymph node at 10× magnification showing extensive non-caseating granulomas (yellow circles) surrounded by lymphocytes. (C) H&E-stained section at 20× magnification highlighting a multinucleated giant cell (red circle) and aggregates of epithelioid histiocytes (black arrows).

A multidisciplinary discussion among hematology-oncology, rheumatology, and infectious disease agreed that a biopsy was crucial for diagnosis. A right submandibular lymph node biopsy revealed extensive non-caseating granulomatous inflammation (Figure 6).

**Figure 7:**
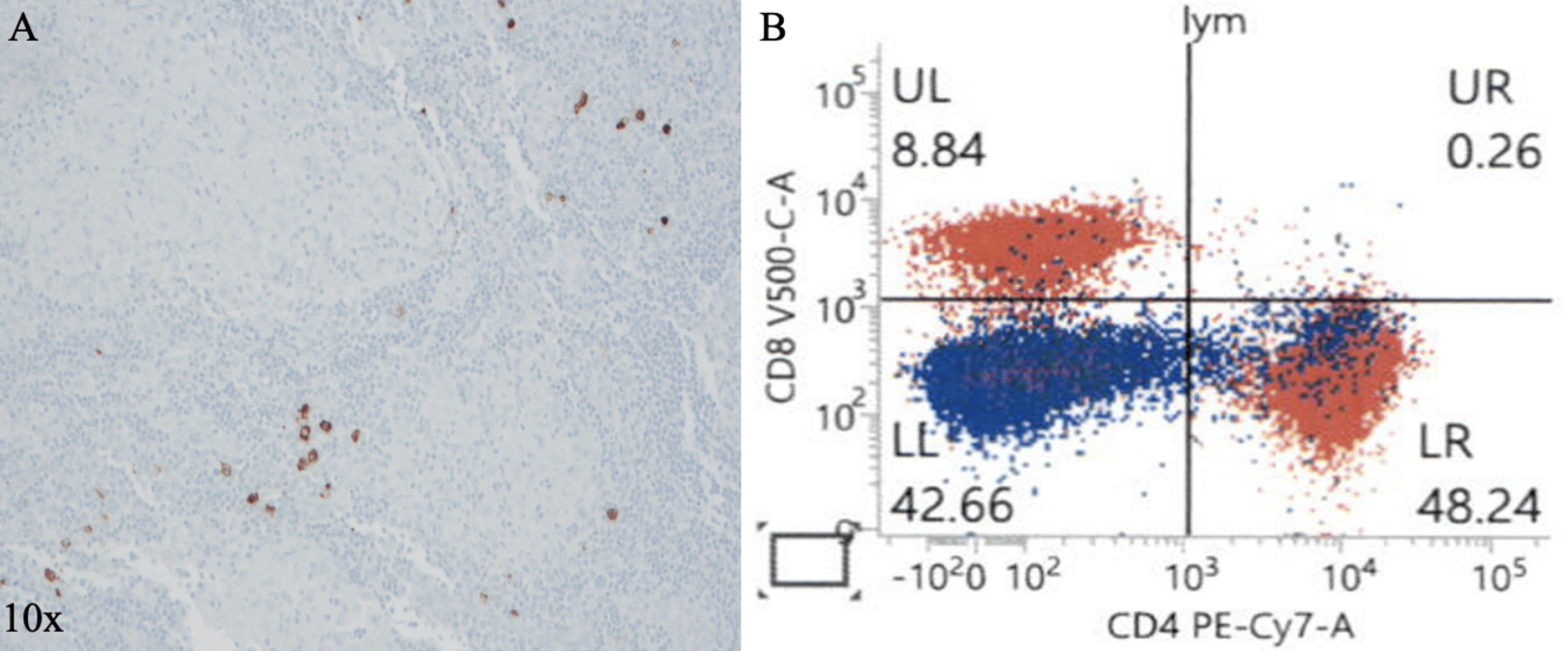
Immunohistochemical and Flow Cytometric Finding (A) IgG4 (10×) staining showing positivity in a small subset of plasma cells. (B) Flow cytometry analysis of lymphocytes, demonstrating 9% CD8+ T-cells (top left) and 48% CD4+ T-cells (bottom right), resulting in a CD4:CD8 ratio >5:1. B-cells are identified as the double-negative population (bottom left).

Immunohistochemistry demonstrated IgG-positive plasma cells with fewer than 10% IgG4-positive cells (Figure 7A), making IgG4-related disease (IgG4-RD) unlikely. Flow cytometry showed no abnormal lymphoid population, but an elevated CD4:CD8 ratio of 5.4 (Figure 7B).

Acid-fast bacilli (AFB) and fungal stains were negative. Based on these findings, sarcoidosis was diagnosed.

The patient received pulse methylprednisolone (1 g IV daily for three days) followed by a tapering oral prednisone regimen (30 mg to 5 mg over 25 days). Given her T1DM and steroid-induced hyperglycemia, methotrexate (25 mg subcutaneously once weekly) and plaquenil (300 mg daily) were added as steroid-sparing therapies, along with folic acid 1 mg daily, except on the methotrexate day. Her calcium level normalized, symptoms resolved, and she was discharged for follow-up with rheumatology and endocrinology (Table 4).

**Table 4:** Follow-Up Laboratory Results (Three-Month Post-discharge) At three-month follow-up, laboratory findings showed normalization of serum calcium and creatinine levels, with improvement in inflammatory markers. The 1,25-dihydroxyvitamin D and ACE levels had decreased significantly, consistent with the resolution of active granulomatous inflammation. ACE, angiotensin-converting enzyme; ESR, erythrocyte sedimentation rate; PTH, parathyroid hormone; RBC, red blood cell; WBC, white blood cell

Category	Laboratory Test	Result	Reference Range
Chemistry profile	Calcium (total)	9.1 mg/dL	8.5-10.7 mg/dL
	Creatinine	0.9 mg/dL	0.6-1.2 mg/dL
	1,25-dihydroxyvitamin D	38.3 pg/mL	19.9-79.3 pg/mL
	PTH	76.3 pg/mL	12.0-65.0 pg/mL
	ACE	81 U/L	18-101 U/L
	Lysozyme	2.08 µg/mL	<4.5 µg/mL
Hematology workup	WBC	11.45 x 10^3^/µL	4.5-13.0 x 10^3^/µL
	RBC	3.67 x 10^6^/µL	4.1-5.1 x 10^6^/µL
	Hemoglobin	10.4 g/dL	12.0-16.0 g/dL
	ESR	27 mm/hr	0-20 mm/hr

## Discussion

Most children who are eventually diagnosed with sarcoidosis initially present with nonspecific constitutional symptoms or may be asymptomatic, making the diagnosis difficult [10]. The abdomen is the most common extrapulmonary site of involvement, with common findings of hepatosplenomegaly and/or abdominal lymphadenopathy, while multiple parenchymal nodules are rare [10]. Our patient showed a strikingly extensive pattern, including multiple splenic nodules, periportal adenopathy, and widespread lymph node enlargement involving cervical, mediastinal, hilar, retroperitoneal, and iliac regions. Additional findings - parotid gland enlargement with intraparotid lymph nodes, small pulmonary nodules with a suspected miliary pattern, and a solitary sternal lytic lesion - represent an exceptionally uncommon combination of manifestations in pediatric sarcoidosis, highlighting the extensive multiorgan nature of the disease in this case.

Sarcoidosis presents with systemic symptoms or metabolic complications, such as hypercalcemia, which is a rare but recognized manifestation, reported in about 6% of adult cases and far less frequently in children [11]. It results from excessive extrarenal production of calcitriol by activated macrophages in granulomas through unregulated 1-α-hydroxylase activity. Excess calcitriol enhances intestinal calcium absorption and bone resorption, leading to hypercalcemia and, in severe cases, nephrocalcinosis or AKI. Prompt recognition of this mechanism was critical, as persistent hypercalcemia can exacerbate renal dysfunction. Renal involvement in pediatric sarcoidosis has been reported in only a few cases [12]. When it occurs, it usually manifests as AKI secondary to hypercalcemia or, less often, granulomatous interstitial nephritis [13]. This patient’s AKI likely resulted from hypercalcemia-induced renal vasoconstriction and dehydration, as renal function rapidly normalized after hydration, indicating a reversible, prerenal process.

A 2021 meta-analysis reported an overall diabetes prevalence of 12.7% among sarcoidosis patients [14,15], though most cases were attributed to steroid use or type 2 diabetes rather than autoimmunity [14,15]. True T1DM in the setting of sarcoidosis has been described only in isolated reports - one probable pediatric case without biopsy confirmation [16] and two adult biopsy-proven cases [9]. The association between sarcoidosis and T1DM likely reflects overlapping immune dysregulation involving the Th1/Th17 pathway, elevated proinflammatory cytokines, and possible genetic susceptibility, such as ACE gene polymorphisms [9,15]. To our knowledge, this represents the first biopsy-confirmed pediatric sarcoidosis coexisting with T1DM, expanding the spectrum of autoimmune and granulomatous disease overlap in childhood.

The main diagnostic challenge in this case was distinguishing sarcoidosis from malignancy and infectious granulomatous diseases. The patient’s nonspecific constitutional symptoms, along with diffuse lymphadenopathy, pulmonary and splenic nodules on CT, raised strong concern for lymphoma, particularly given her background of T1DM. Both conditions can present with lymphadenopathy and non-caseating granulomas, making distinction difficult and chronic immune activation in sarcoidosis has been proposed to predispose to lymphoid proliferation [8]. The patient’s animal contact history suggested possible infectious etiologies. However, comprehensive infectious testing was negative. Lymph node biopsy showed extensive non-caseating granulomatous inflammation (Figure 6) with an elevated CD4:CD8 ratio (5.4) and no abnormal lymphoid population on flow cytometry, excluding lymphoma (Figure 7B). Limited IgG4 expression (<10%) on special stains made IgG4-RD unlikely (Figure 7A). Together, these findings established the diagnosis of sarcoidosis, which in this case closely mimicked both infectious and malignant conditions.

Management of sarcoidosis depends on disease severity and organ involvement. In high-risk cases with multiorgan disease or metabolic complications such as hypercalcemia, early immunosuppressive therapy is essential to control inflammation and prevent further organ injury. Systemic corticosteroids remain the first-line treatment [17]. Our patient received high-dose IV methylprednisolone for rapid suppression of granulomatous activity, followed by a tapering course of oral prednisone to maintain remission and minimize steroid-related side effects. Low-dose methotrexate and plaquenil were added as steroid-sparing agents to support long-term disease control and reduce steroid exposure, with folic acid to prevent adverse effects. This regimen follows current pediatric sarcoidosis management recommendations and was effective in achieving remission in this high-risk case [17].

## Conclusions

This case describes a 16-year-old girl with T1DM who presented with severe hypercalcemia, AKI, and widespread lymphadenopathy, ultimately confirmed as having sarcoidosis with multiorgan involvement. The combination of pediatric onset, autoimmune background, and extensive systemic disease makes this case unique and diagnostically challenging. Sarcoidosis should be considered in children with PTH-independent hypercalcemia and lymphadenopathy after infectious and malignant causes are excluded, with histopathologic confirmation remaining essential. The coexistence of sarcoidosis and T1DM, though rare, may reflect shared autoimmune mechanisms. Multidisciplinary collaboration and timely immunosuppressive treatment were key to accurate diagnosis and achieving stabilization.
